# Some Cases at the Society for the Study of Disease in Children

**Published:** 1906-03-24

**Authors:** 


### Hospital Clinics.

### SOME CASES AT THE SOCIETY FOR THE STUDY OF DISEASE IN CHILDREN.

Many of the cases shown at the meeting of this Society on Friday, March 16, were of unusual interest and value. Among these must be placed an example of precocious development, shown by Mr. Hugh Lett. The subject was a boy of four years, whose general appearance, height, and muscular development suggested a growth of thirteen or fourteen years, whilst the voice, the growth of pubic hair, and the size of the external genitals were comparable to those usually found at sixteen or seventeen years of age. Until two years old the boy had appeared to be of ordinary growth and development, but after that date he rapidly assumed his present characters. Reference was made to the fact that a certain number of these cases had been found to be associated with tumours of the suprarenal bodies, and occasionally with other abdominal growths; but in the present case no evidence of any such condition could be discovered. In this connection a very interesting experience was quoted by Mr. Clement Lucas. This was the case of a female child in whom precocious development of the genitals, mammæ, and other parts was found together with a solid tumour of the ovary, and upon the removal of this there was a general retreat of the abnormal development, so that but for the persistence of a quantity of pubic hair the child became exactly like other girls of her age. The influence of tumours in various situations, but chiefly in the abdomen, in determining abnormal growth is beyond question, though how this is exercised is difficult to explain. On the other hand, as in the present instance, such cases occur apart from any discoverable tumour, and so afford ground for a suggestion made by Dr. Parkes Weber, that precocious development is an example of reversion to a lower type where full development is reached without the prolonged infancy and childhood of man. As regards the effects of tumour in determining abnormal growth it may be remembered that there is at least one case on record where a localised hypertrophy of one lower limb was found to be associated with a tumour in the optic thalamus.

A second case shown by Mr. Hugh Lett raised a point of great practical importance in reference to treatment. The patient was an infant showing an "obstetrical paralysis" of the right upper limb (Erb-Duchenne paralysis), and the question was whether the treatment should be rest by means of a splint and the regular use of massage, or whether an operation should be performed with a view to repair the damaged nerve structures. There was a general agreement that in this particular instance the former was the proper course (though some urged that the splint should now be discontinued), as there had been evidence of decided improvement. But on the general prognosis of such cases there was much difference of opinion. By some speakers it was held that most, if not all, of these cases made a good recovery, and it was argued in support of this, that persistence of the paralysis in adults was practically never seen. On the other hand, it was urged that this was too roseate a view. Duchenne, it may be noted, held that the prognosis should be "most guarded," and Dr. James Taylor, in his recent book on "Nervous Diseases in Childhood," pronounces it to be "bad. . . . Complete recovery," he says, "is very rare." There can be no question that in a number of cases operation has produced striking results, largely due to the work of Dr. Robert Kennedy, of Glasgow.[1]

A third case was one of coloboma of the upper eyelid, and was shown by Mr. Sydney Stephenson. It was introduced with a discussion of the etiology of the condition and with a suggestion that as the conventional descriptions of the development of the eyelid did not explain the occurrence of the coloboma, it was probable that they needed to be scrutinised and revised. A more practical point was the advice that, apart from the æsthetic aspect, an operation should be advised in order to secure adequate protection for the eyeball. In this connection Mr. Stephenson related a case in which, together with a coloboma of the lid, was found a perforating ulcer of the cornea, which led in the end to loss of the eyeball. It seems reasonable to believe that the imperfect protection of the cornea which a coloboma involves may be responsible for these unfortunate developments.

A characteristic case of achondroplasia was shown by Dr. Kenneth R. Hay, and excited general interest. A number of cases of this condition have now been figured, so that it can hardly escape recognition. It has been suggested that thyroid gland might be tried as a remedy, but, as Dr. Cautley remarked in the course of the discussion, the condition as seen after birth is not an active disease but the result of disease occurring in early intra-uterine life. Hence medication of any kind can hardly be expected to prove of value. Mr. G. Pernet has drawn attention to the fact that in the British Museum there are a number of statuettes showing the deformities of achondroplasia, and there seems reason to believe that they were objects of veneration or worship.[2]

Another question which engaged the attention of the Society was the treatment of congenital hypertrophic stenosis of the pylorus. It arose on a specimen of this condition shown by Dr. Edmund Cautley, and taken from the body of an infant weighing 3½ pounds. Dr. Cautley expressed a robust confidence in the virtues of surgical operation, and contended that the prognosis of operation was determined by the general state of the child. If the question had not been too long postponed, and the child thus allowed to get into a wasted and ill-nourished state, the prospects were extremely good. On the other hand, prolonged delay, with its necessary prejudice to the child's general condition, meant a corresponding diminution of the chances of success. Hence it seemed obvious that the proper proceeding, when the diagnosis is once made, is to advise that the child be submitted to operation.

No one present at the meeting actively dissented from Dr. Cautley's view, but that there is another side to the question can be seen from an article by Dr. Geo. F. Still which appeared in the *Lancet* (March 11, 1905).

Two pathological specimens of interest were shown by Dr. C. O. Hawthorne. The first was a tumour of the pons taken from the body of a boy of eight years. The clinical history illustrated how readily the diagnosis of intracranial growths may be overlooked unless the ophthalmoscope is used as a routine practice. In this case, and the experience is not an uncommon one, the boy had for two to three months had frequent headaches and sometimes attacks of vomiting. But he had never been regarded as seriously ill. Afterwards he began to show a number of jerking movements, and was thought to have St. Vitus's dance. Then he was brought to hospital, and, on examination, was found to have marked double optic neuritis—a fact which, in the circumstances, placed the diagnosis beyond question. It was noteworthy that a marked clinical feature of the case was considerable opisthotonos with retraction of the head. Another noteworthy fact was the absence of ocular paralysis or other distinctive evidence of the position of the tumour. The second specimen shown by Dr. Hawthorne included the kidneys and heart taken from the body of a girl of twelve years who, during life, had been the subject of albuminuria (without dropsy), cardiac hypertrophy, and neuro-retinitis. Here again it was pointed out the diagnosis might have been missed but for the use of the ophthalmoscope, as the girl complained merely of headache and loss of flesh. Attention was directed to the physical characters of the kidneys, and it was suggested that they corresponded to the so-called small white kidney rather than to the granular red variety. It was contended that the most suitable clinical name for the condition would be "chronic renal disease without dropsy," in preference to chronic interstitial nephritis, which latter suggested relationships that had not been proved to exist. The importance of the case to the practitioner lies in its illustration of the fact that chronic disease of the kidneys of a most serious type may exist in children without dropsy and without recognised urinary disturbances. Quite a number of cases of this type have now been published by Dr. Leonard Guthrie, Dr. Geo. A. Sutherland, Dr. Jas. Sawyer, Dr. Geo. Carpenter, and others.

[1] Brit. Med. Jour., 1903 and 1904. [2] Brit. Jour. of Chil. Dis., Jan. 1904.

